# Avulsion fracture of the medial head of the gastrocnemius muscle associated with multiple ligament injuries before closure of the growth plate: a case report

**DOI:** 10.1186/s13256-019-2325-z

**Published:** 2019-12-25

**Authors:** Masataka Hirotsu, Hironori Kakoi, Noboru Taniguchi

**Affiliations:** Department of Orthopaedic Surgery, Graduate School of Medical and Dental Sciences, Kagoshima University, 8-35-1 Sakuragaoka, Kagoshima-city, Kagoshima 890-8520 Japan

**Keywords:** Avulsion fracture, Medial head of the gastrocnemius muscle, Growth plate, Posterior cruciate ligament

## Abstract

**Background:**

Avulsion fracture of the medial head of the gastrocnemius muscle is a very rare phenomenon. There are no reports of avulsion fractures associated with multiple ligament injuries before closure of the growth plate. The authors present a case of avulsion fracture of the insertion of the medial head of the gastrocnemius muscle associated with posterior cruciate ligament injury and an avulsion fracture of the medial collateral ligament at the femoral attachment.

**Case presentation:**

A 15-year-old Japanese boy was injured by contact with another player while playing soccer. He was immediately admitted to the authors’ hospital with knee pain. Radiography and computed tomography revealed an avulsion fracture of the medial collateral ligament at the femoral attachment and an avulsion fracture of the medial head of the gastrocnemius muscle. In examination under anesthesia, the Lachman test was negative and posterior drawer test was positive. Fixation of the avulsion fractures of the medial collateral ligament and medial head of the gastrocnemius was performed 9 days after the injury. After fixation, valgus instability at full extension had disappeared. The knee was immobilized in a brace for 2 weeks postoperatively. One year postoperatively, the posterior drawer test was slightly positive; however, our patient was able to ambulate without pain and returned to sports without feeling instability in his knee.

**Conclusion:**

A rare case of avulsion fracture of the gastrocnemius muscle combined with multiple ligament injuries before closure of the growth plate is described. A satisfactory result was obtained by fixation of the avulsed bone fragments of the gastrocnemius muscle and medial collateral ligament. The authors believe that avulsion fracture of the medial head of the gastrocnemius muscle associated with posterior cruciate ligament injury should be repaired.

## Background

Avulsion fracture of the medial head of the gastrocnemius muscle is a very rare phenomenon; to the best of our knowledge, only a few cases have been reported to date. Two of these reports described isolated avulsion fractures of the medial head of the gastrocnemius muscle. Another case involved a 51-year-old man who sustained multiple ligament injuries and posterior dislocation of his knee. There have, however, been no reports of avulsion fracture combined with multiple ligament injuries before closure of the growth plate. If this fracture is not recognized, it may be missed. We present a case of avulsion fracture of the insertion of the medial head of the gastrocnemius muscle associated with posterior cruciate ligament (PCL) injury and avulsion fracture of the medial collateral ligament (MCL) at the femoral attachment.

## Case presentation

**Fig. 1:**
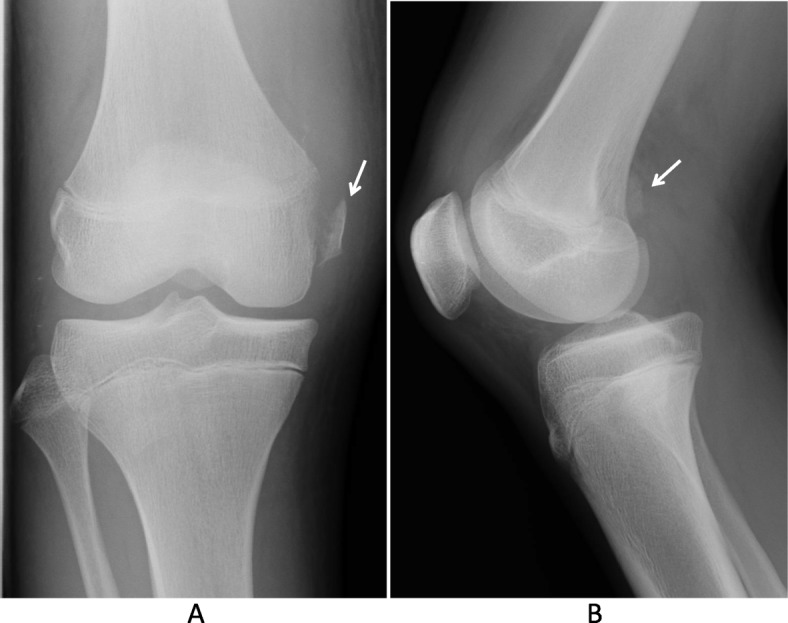
**a** and **b** Plain radiographs of the injured knee. Anteroposterior (**a**) and lateral (**b**) radiographs showing an avulsion fracture of the medial collateral ligament (*arrow*, **a**) and a bone fragment in the popliteal space (*arrow*, **b**)

**Fig. 2:**
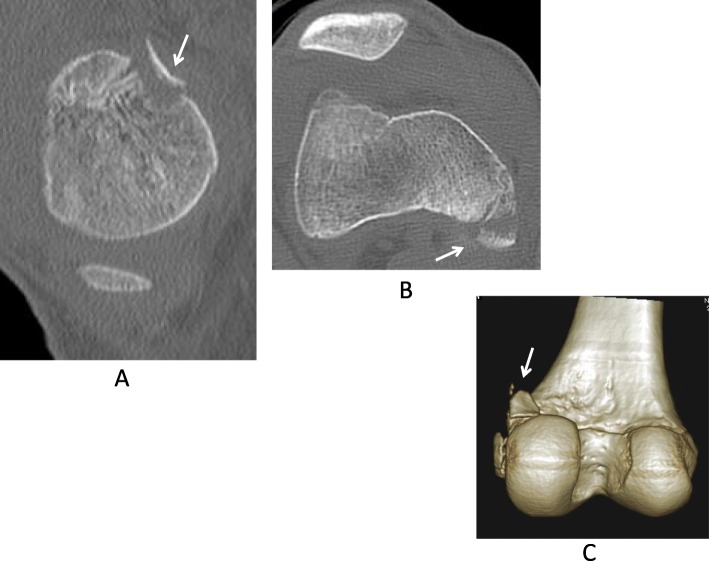
**a** and **b** and **c** Computed tomography of the injured knee. An avulsion fracture of the medial head of the gastrocnemius muscle in the sagittal plane (*arrow*, **a**), and in the axial plane (*arrow*, **b**), and in the three-dimensional computed tomography (*arrow*, **c**)

**Fig. 3:**
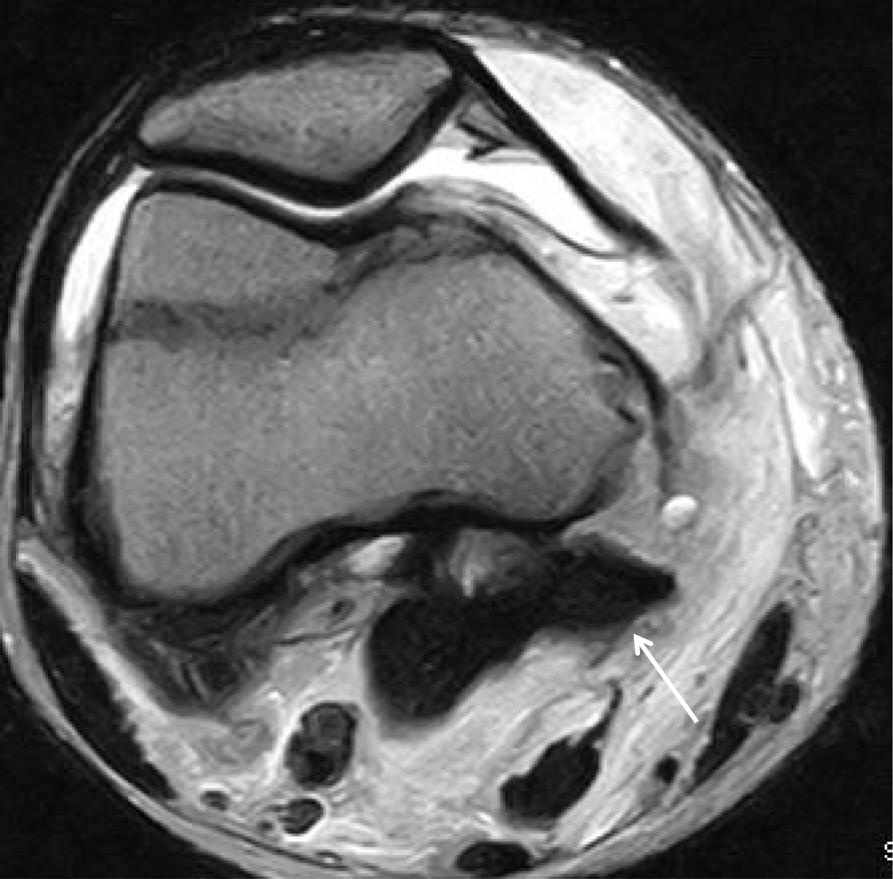
Magnetic resonance imaging of the injured knee. The axial view shows the bone fragment attached to the medial head of the gastrocnemius muscle (*arrow*)

A 15-year-old Japanese boy was injured by contact with another player while playing soccer. He was immediately admitted to the authors’ hospital with knee pain. Anteroposterior radiographs revealed avulsion fracture of the MCL at the femoral attachment (Fig. 1a). Lateral radiographs revealed posterior sagging of his tibia and bone fragments in the posterior aspect of the femoral condyle (Fig. 1b). Computed tomography revealed avulsion fracture of the medial head of the gastrocnemius muscle (Fig. 2a–c). Although magnetic resonance imaging (MRI) revealed continuity of the PCL, a high-intensity signal change of the femoral footprint of the PCL was recognized in T2-weighted images. MRI also revealed a bone bruise of the lateral femoral condyle and lateral tibial condyle. The medial head of the gastrocnemius muscle and a detached bone fragment were continuous (Fig. 3), while the meniscus remained intact.

**Fig.4:**
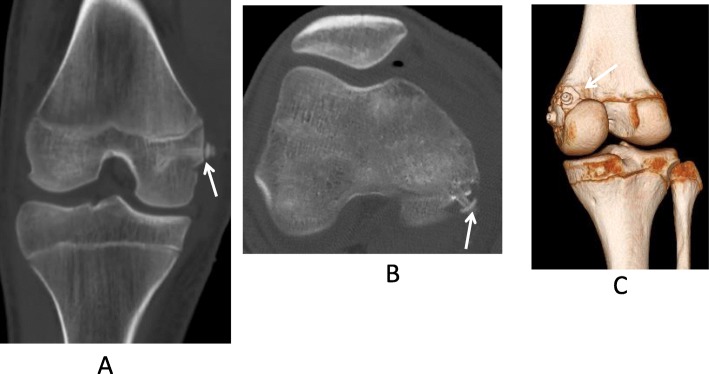
**a** and **b** and **c** Computed tomography of the injured knee after surgery. An avulsion fracture of the medial collateral ligament was reduced and fixed with an absorbable screw (*arrow*, **a**). An avulsion fracture of the medial head of the gastrocnemius muscle was reduced and fixed with an absorbable screw in the axial plane (*arrow*, **b**), and in three-dimensional computed tomography (*arrow*, **c**)

**Fig. 5:**
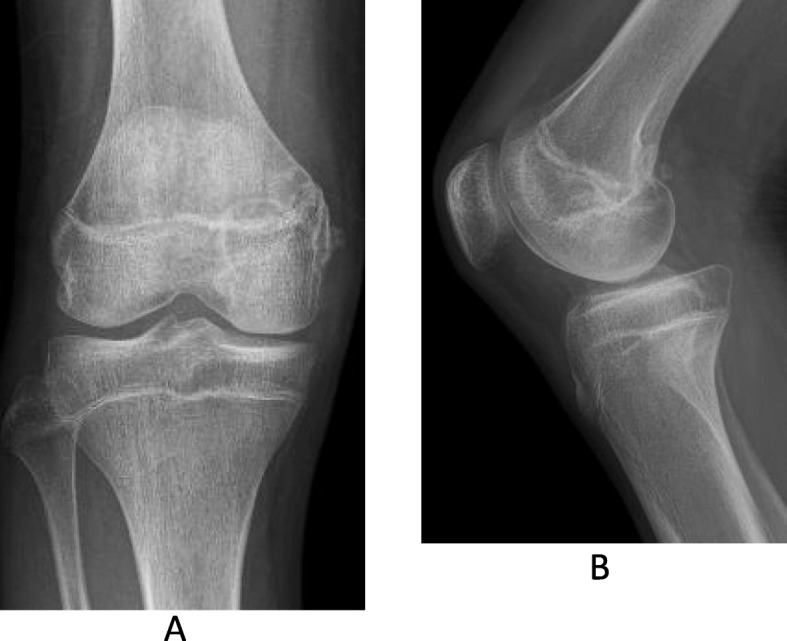
**a** and **b** Plain radiographs of the injured knee 3 months after surgery. Anteroposterior (**a**) and lateral (**b**) radiographs showing solid osseous healing of the avulsion fracture of the medial collateral ligament and the avulsion fracture of the medial head of the gastrocnemius muscle

Fixation of the avulsion fracture of the MCL and of the medial head of the gastrocnemius muscle was performed 9 days after injury. In examination under anesthesia, the Lachman test was negative and the posterior drawer test was positive. In arthroscopic findings, intra-articular hematoma was present, and the anterior cruciate ligament (ACL) remained intact. Continuity of the PCL fibers was apparent; however, at the femoral attachment of the PCL, bleeding was recognized and the tension of the PCL was slightly loose on probing. A medial skin incision was made around the bone fragment of the MCL. The bone fragments of the gastrocnemius muscle and MCL were reduced and fixed using an absorbable screw (diameter 4.5 mm) and a washer (Fig. 4a, b, c). After fixation, valgus instability at full extension had disappeared. Our patient’s knee was immobilized in a brace for 2 weeks postoperatively. Range-of-motion exercises were started 2 weeks after surgery. Partial weight bearing was started using a PCL brace 6 weeks after surgery. He wore a PCL brace for 3 months. Three months after surgery, radiographs revealed solid osseous healing of both the avulsion fractures (Fig. 5a, b). One year postoperatively, the posterior drawer test was slightly positive; however, our patient was able to ambulate without pain and returned to sports without feeling instability in his knee.

## Discussion and conclusion

Avulsion fracture of the medial head of the gastrocnemius muscle is a very rare phenomenon; to the best of our knowledge, only three cases have been reported to date. Maehara and Sakaguchi [1] reported a case of isolated avulsion fracture without ligament injury. The patient sustained an avulsion fracture in a skiing accident and underwent surgery to repair the bone fragment using a cancellous screw. Patterson *et al.* [2] reported satisfactory results using conservative treatment for an isolated avulsion fracture in a 14-year-old man, who was a wrestler and returned to competition. Mio *et al*. [3] reported a case involving a 51-year-old man with an avulsion fracture associated with multiple ligament injuries and posterior dislocation of the knee in a high-energy traffic accident. The avulsion fracture of the medial head of the gastrocnemius muscle was combined with ACL, PCL, and MCL injuries. The MCL was avulsed from the tibial attachment and reattached to its tibial insertion using suture anchors. The avulsion fracture of the medial head of the gastrocnemius muscle was reduced and fixed using a screw.

Our case involved a sports injury and an avulsion fracture combined with multiple ligament injuries before closure of the growth plate. We suspected that the knee had severe valgus stress, same as in the case described by Mio *et al.*, [3] and the avulsion fracture occurred at the femoral attachment of the MCL, which is a weak part in mechanical strength. Regarding whether to repair an avulsed fragment, Maehara and Sakaguchi [1] reported that the case was an isolated fracture; however, the authors chose surgery to address the large displaced bone fragment. In a case reported by Patterson *et al.* [2], a fracture was found 4 weeks after injury, and there was no instability of the knee and only limited range of motion. As such, they did not perform surgery, and the patient returned to competition 8 weeks after injury. On the other hand, Mio *et al*. [3] reported that they initially repaired the avulsed bone fragment of the gastrocnemius muscle and repaired the MCL and planned to reconstruct the ACL and PCL. They also reported that, although the posterior dislocation of the knee was easily reduced manually, the knee easily dislocated again because of gross instability; however, the reduced position could be maintained by repairing the MCL and gastrocnemius muscles. Inoue *et al.* [4] performed electromyographic analyses in 12 patients with PCL-deficient knees to compare electrical activity in the quadriceps, hamstring, and gastrocnemius muscles between the uninjured and involved knees. The authors reported that before generation of flexion torque, the gastrocnemius muscle was significantly electrically activated earlier in the PCL-deficient knees than in uninjured knees. They suggested that early contraction of the gastrocnemius muscle may be part of a compensatory mechanism in PCL-deficient knees. Mio *et al.* [3] also reported that reduction of the bone fragment attached to the medial head of the gastrocnemius muscle was crucially important to regain stability of the knee. Similarly, although slight posterior instability after surgery remained in our case, we speculate that fixation of the avulsion fracture of the MCL and gastrocnemius muscle may have contributed to posterior stability. Therefore, we believe that the avulsion fracture of the medial head of the gastrocnemius muscle associated with PCL injury should be repaired. Regarding the mechanism of injury, Arner and Lindholm [5] reported that rupture of the gastrocnemius muscle probably occurs with sudden dorsiflexion of the foot with the knee joint in extension. Maehara and Sakaguchi [1] suggested that the mechanism of avulsion fracture of the gastrocnemius muscle may be sudden dorsiflexion of the foot with full extension of the knee. Mio *et al*. [3] suspected that the mechanism of injury may be hyperextension and severe valgus stress on the knee and increased mechanical torsion to the medial head of the gastrocnemius muscle by knee hyperextension, and that contraction of the gastrocnemius muscle likely produced the avulsion fracture. Considering these reports, we believe that the mechanism of injury in our case involved hyperextension and valgus stress on the knee and sudden dorsiflexion of the foot. As a result, we considered that the avulsion fracture occurred at the femoral attachment of the MCL, which is a weak part in mechanical strength and at the insertion of the medial head of the gastrocnemius muscle. Regarding the material used for fixation, we used an absorbable screw so that it did not have to be removed. Because bone union was achieved 1 year after surgery, there was no problem in terms of strength, and we considered the absorbable screw as a suitable option for fixation.

We described a rare case of avulsion fracture of the gastrocnemius muscle combined with multiple ligament injuries before closure of the growth plate. A satisfactory result was obtained by fixation of the avulsed bone fragments of the gastrocnemius muscle and MCL. We believe that the avulsion fracture of the medial head of the gastrocnemius muscle associated with PCL injury should be repaired.

## Data Availability

Not applicable.

## Abbreviations

ACL: Anterior cruciate ligament
MCL: Medial collateral ligament
MRI: Magnetic resonance imaging
PCL: Posterior cruciate ligament
